# Associations between Parental and Friend Social Support and Children's Physical Activity and Time Spent outside Playing

**DOI:** 10.1155/2017/7582398

**Published:** 2017-02-28

**Authors:** Constantinos A. Loucaides, Niki Tsangaridou

**Affiliations:** ^1^Ministry of Education and Culture, 1434 Nicosia, Cyprus; ^2^Department of Education, University of Cyprus, P.O. Box 20537, Nicosia, Cyprus

## Abstract

The purpose of this study was to examine the structural validity of a parent and a child questionnaire that assessed parental and friends' influences on children's physical activity and investigate the associations between the derived factors, physical activity, and time spent outside. Children (*N* = 154, mean age = 11.7) and 144 of their parents completed questionnaires assessing parental and friends' influences on children's physical activity. Children wore a pedometer for six days. Exploratory factor analyses revealed four factors for the parental and five for the child's questionnaire that explained 66.71% and 63.85% of the variance, respectively. Five factors were significantly associated with physical activity and five significantly associated with time spent outside. Higher correlations were revealed between “general friend support,” “friends' activity norms,” and physical activity (*r* = 0.343 and 0.333 resp., *p* < 0.001) and between “general friend support” and time spent outside (*r* = 0.460, *p* < 0.001). Obtaining information relating to parental and friends' influences on physical activity from both parents and children may provide a more complete picture of influences. Parents and friends seem to influence children's physical activity behavior and time spent outside, but friends' influences may have a stronger impact on children's behaviors.

## 1. Introduction

Research indicates that participation in physical activity (PA) results in beneficial effects on children's and adolescents' musculoskeletal health, blood pressure, cardiovascular risk factors, and overweight and obesity [1, 2]. In addition, recent findings suggest that leisure time PA has a meaningful association with life expectancy [3]. There is an urgent need for promoting PA among children in Cyprus, as data indicate that there has been an increase in the percentage of overweight and obese children from 22.4% in 2000 to 28.2% in 2010 [4]. Furthermore, in a study conducted among children in eight European countries [5], only 2.0% of girls and 20.1% of boys in Cyprus appear to achieve the recommended 60 minutes of moderate to vigorous PA per day. A first step in promoting PA is to understand the correlates of PA behavior that will help inform, develop, and improve the effectiveness of intervention studies [6].

An important correlate of children's PA is parental support, as a recent meta-analysis showed that the association between parental support and child's PA yields a medium effect size [7]. Review studies identified parental modeling and tangible and intangible support as potential parental correlates of children's PA [7–10]. While review studies identified a number of parental influences on children's PA, they argue that future research needs to also look into other significant people in children's immediate social network including friends [7, 10, 11]. Friends have been found to influence children's PA levels through a number of processes including communication such as encouragement, modeling through their own behavior, and participating in PA with friends [12, 13].

Because of the significant influence that parents and friends exert on children's PA levels, studies should address both of these factors when assessing correlates of PA. A study among 10–12-year-old European children found that the joint or simultaneous effects of both of these sources of support are important in promoting health behaviors [14]. Examining the mechanisms by which parents and friends influence children's PA levels is especially important, as evidence suggests that there is a significant decline in these two sources of support from year 7 and year 9 [15] and low social support for PA in early adolescence appears to be tracking into later adolescence [16].

Studies have also examined parental effects on children's time spent outside. Social support by parents [17] and parental encouragement [18] have been found to be associated with time spent outside. A limitation of these studies is that they did not examine the association between friends' influences on time spent outside. One study showed that time spent outside with friends makes the greatest contribution to children's PA [19], but still little is known about who children spend their time with after school and how this relates to their PA levels [19]. Understanding how parents and friends influence children's time spent outside is important, as outdoor time has been consistently associated with children's PA levels [20, 21].

Interestingly, while a number of studies examined parental and friends' influences on children' PA, these studies obtained information either from parents only [17, 18, 22, 23] or from children only [15, 16, 24–27], with no studies obtaining information from both parents and children. By administering instruments measuring the same constructs to both children and parents, researchers can investigate whether parents and children perceive parental practices in different ways, and how these perceptions correlate with PA behavior [28]. Collecting similar data from different sources may help to further extent our knowledge of parental and friends' influences on children's PA.

In Cyprus, if we are to promote PA levels among children and help combat the rising prevalence of overweight and obesity, we need to develop valid and reliable measures that examine influences of PA for use in correlational studies as well as in intervention studies to examine program effectiveness. This will also add evidence in the international literature, as the majority of published studies used measures of indeterminate validity and reliability, cited studies that have validated the measures in different populations and did not adequately describe the items and response format of the measures used in the population under study [29, 30]. Calls have been made for the development of more multidimensional, comprehensive measures of PA parenting that provide evidence of validity and reliability [30].

Therefore, the purpose of the present study was threefold: (1) to provide evidence of validity and reliability of a parental and a child questionnaire assessing parental and friends' influences on children's PA, (2) to examine potential associations between parental and friends' factors derived from the two questionnaires and children's PA and time spent outside playing, and (3) to investigate with whom children spent time out and how this relates to their PA levels.

## 2. Methods

### 2.1. Participants

Data from this study are part of a larger study that examined correlates of PA and sedentary behavior among children in Cyprus. All thirteen elementary schools from the town of Paphos were invited to participate in the study and six schools agreed to participate. Informed consent was sent to all parents of eleven-year-old and twelve-year-old children in these schools and 154 children (53.5%) agreed to participate by returning signed informed parental/child consent. Ethical approval for this study was granted by the Centre of Educational Research and Evaluation of the Cyprus Ministry of Education.

### 2.2. Parental and Child Questionnaires

Two questionnaires, one for parents and one for their children, were constructed to examine correlates of children's PA. Items from these questionnaires were written based on three sources: from previous qualitative [31] and quantitative work [32] of factors influencing Cypriot children's PA levels and from other studies that used scales to assess social influences on children's PA participation [23, 24, 33, 34]. In total, 15 items were written for the parental questionnaire and 19 items for the child questionnaire. Of those items, 10 were common to both questionnaires and assessed parental modeling (parental participation and parent-child coparticipation in PA) adopted from the Jago et al. [24] study and parental tangible/logistic and intangible support for their child's PA adopted from a number of previous studies [23, 24, 33, 34].

The remaining five items in the parental questionnaire assessed the existence of friends and friends' houses in the neighborhood and whether the child meets friends, visits friends' houses in the afternoon to play, or attends a sports club with a friend. All responses in the parental questionnaire were scored on a four-point scale ranging from strongly disagree to strongly agree with the exception of the two items assessing daily parental participation in PA that were assessed with a seven-point scale with responses including “not at all,” “up to 30 min,” “up to one hour,” “up to two hours,” “up to three hours,” “up to four hours,” and “more than 4 hours.” Nine items in the child's questionnaire assessed friends' influences on PA. Six items assessed the weekly frequency of six behaviors: whether the child's best friend attends a sports club; whether the child attends a sports club with his/her best friends; whether the child visits the house of a friend to play; whether the child engages in physical activity with friends in the afternoon; whether the child's friends go outside in the afternoon to play a sport; and whether the child's friends encourage him/her to engage in PA. These items were scored on a six-point scale with responses including “not at all,” “once,” “two times,” “three times,” “four times,” and “more than four times.” Three items on a four-point scale ranging from strongly disagree to strongly agree assessed friends' participation in PA and norms about PA adopted from the Jago et al. study [24].

### 2.3. Physical Activity and Time Spent outside Measurement

Children's PA was monitored for six days (including four school-days and two weekend days) using the spring-levered DW-200 YAMAX pedometer (Yamax Corporation, Tokyo, Japan). This pedometer is the most widely used in surveillance studies and has been shown to be valid and reliable among children [35, 36]. Furthermore, its low cost and ease of use make it an appropriate instrument for measuring PA in low-budget studies. For each day that children wore the pedometer, they also completed a diary relating to the time that they spent outside the house playing. Time spent outside was scored on a seven-point scale with response options including “not at all,” “up to 30 minutes,” “up to 1 hour,” “up to 2 hours,” “up to 3 hours,” “up to 4 hours,” and “more than four hours.” For deriving time spent outside, each point of the scale was assigned its corresponding numeric value (e.g., “not at all” = 0 minutes, “up to 30 minutes” = 30 minutes, and “up to 1 hour” = 60 minutes). Children also noted with whom they spent each day outside playing by choosing from five different options including “alone,” “brothers or sisters,” “friend(s),” “parents,” and “other adult.” Similar diaries were used in previous studies in children in the UK [19] and Cyprus [37].

### 2.4. Procedures

Two research assistants visited each school to administer the questionnaires, instruct the children on how to use the pedometer, and measure children's weight and height in the presence of school teachers. Children's height and weight for the calculation of the Body Mass Index (BMI, kg/m^2^) were measured using a portable stadiometer (SECA 220, Hamburg-Germany) and digital scale (SECA 767, Hamburg-Germany). The parental questionnaire was given to the children to take home for completion by their parents. Pedometers were given to the children one day prior to the monitoring period for familiarization. Children were instructed to fit and reset the pedometer at their waist in the morning before coming to school and remove the pedometer at night before going to sleep (except when bathing or swimming). Children were given recording cards and recorded their step counts at the end of the school day (13:05) and just before they went to bed. During the weekend days, children recorded their steps once per day, before they went to bed. For each day, children were also requested to complete the diary relating to the time that they spent outside the house.

### 2.5. Statistical Analyses

Descriptive statistics including means and standard deviations and frequencies and percentages were computed to describe the sample's characteristics. Two principal component analyses using orthogonal rotation (Varimax) were conducted: one on the 15 items included in the parental questionnaire and one on the 19 items included in the children's questionnaire. A combination of three criteria was utilized for the number of factors to retain that included eigenvalues greater than one, inspection of the scree plot [38–40], and a solution that accounts for a minimum of 60% of the total variance [40]. Based on the guidelines by Tabachnick and Fidell [38], items with factor loadings above 0.32 were included in scales as they approximate 10% overlapping variance between the item and the factor. Items with loadings above 0.32 on more than one item (cross loadings) were considered for deletion, especially if there were other items with higher loadings on each factor [39]. Since this study is exploratory, summated scales were constructed rather than factor scores, as the former are more stable across samples and can be easily replicated across studies [40, 41]. Cronbach's alpha coefficients were computed for each of the scales.

Pearson's correlation coefficients were then computed to examine the associations between the derived scales from both questionnaires as well as the associations between the derived scales, pedometer-determined PA, and time spent outside playing. Correlation coefficients ranging from 0.10 to 0.29, from 0.30 to 0.49, and from 0.50 to 1.0 are considered of low, moderate, and high strength, respectively [42]. Mean values for weekday steps and weekday time spent outside were derived by averaging the measurements across the four weekdays and, for the weekend, by averaging the data obtained during the two weekend days. Means and 95% confidence intervals were computed for PA and time spent outside for each of the monitored days, across three categories of person with whom the child reported spending time out. Because of the small sample size, 95% confidence intervals are calculated so that readers are better able to interpret the strength of the evidence presented [43]. Finally, one-way ANOVAs were conducted to examine whether PA and time spent outside differed across three categories of person with whom the child reported spending time out.

## 3. Results

Out of the 154 children who participated in the study, 61 or 39.6% were boys and 93 or 60.4% were girls. Age of participants (Mean ± SD) was 11.7 ± 0.6 and BMI was 20.3 ± 4.2. Out of the 144 parents who completed the questionnaire, 27 or 18.8% were men and 117 or 81.3% were women.

### 3.1. Structural Validity and Reliability of the Scales

The initial principal component analysis on the 15 items of the parental questionnaire revealed two items that loaded on two different factors. The item “I encourage my child to be physically active” had cross loadings of 0.352 and 0.664, and the item “I watch my child when s/he is engaging in PA” had cross loadings of 0.380 and 0.520. As these loadings exceeded the 0.32 threshold and other items had higher loadings on the factors [38, 39], they were candidates for deletion [39, 40]. The item “I watch my child when s/he is engaging in PA” was removed, but the item “I encourage my child to be physically active” was retained because of its importance to the research objective [40]. In the new factor model cross loadings persisted, and this item was also removed from the model. The cross loadings could be explained by the associations between the variable “I encourage my child to be physically active” and the variables “I talk to my child about the benefits of physical activity” (*r* = 0.580, *p* < 0.001), and “I pay for my child to attend a sports club” (*r* = 0.527, *p* < 0.001) that were retained in the analysis.

The remaining 13 items resulted in the extraction of four factors that explained 66.71% of the variance, KMO = .70, Bartlett's Test of Sphericity *χ*^2^ (78) = 613.88, *p* = 0.001. Five items loaded on factor one that concerned parental support for PA that included both tangible/logistic support and intangible support and was therefore called “general parental support.” Four items loaded on factor two that included statements relating to whether the child meets friends in the afternoon to play and existence of friends in the neighborhood and was therefore named “neighborhood play with friends.” Two items were loaded on each of the last two factors, with the first one including items relating to parental PA participation during weekdays and weekends and was therefore named “parental PA” and the second one including items relating to parental PA participation with child during weekdays and weekends and was named “parental PA with child.” Table 1 presents factor loadings after rotation, eigenvalues, percentage of variance explained by each factor, and Cronbach's alpha for each scale. Reliability of the scales ranged from 0.828 to 0.650.

The principal components analysis (19 items) that was performed on the children's questionnaire also revealed two items that loaded on two different factors. The item “I attend a sports club with a friend” had cross loadings of 0.430 and 0.619 and the item “my best friend attends a sports club” had cross loadings of 0.416 and 0.525. As these loadings exceeded the 0.32 threshold and other items had higher loadings on the factors [38, 39], they were removed from the model [39, 40]. The analysis on the remaining 17 items resulted in five factors that explained 63.85% of the variance, KMO = .78, Bartlett's Test of Sphericity *χ*^2^ (136) = 773.68, *p* = 0.001. Four items that loaded on factor one included statements relating to parental PA participation and parental coparticipation with the child during weekdays and weekends and consequently was called “active parents.” Factor two also included four items relating to encouragement from friends, coparticipation in PA with friends, and friend PA participation and was therefore named “general friend support.” Four items were also loaded on factor three that included parental tangible/logistic support and so was named “tangible parental support.” The fourth factor included items relating to friends' norms about PA participation and friends' PA participation and was hence called “friends' PA norms.” The fifth factor included two items relating to parental encouragement and communicating the benefits of PA to the child and was therefore called “intangible parental support.” Table 2 presents factor loadings after rotation, eigenvalues, percentage of variance explained by each factor, and Cronbach's alpha for each scale. Reliability of the scales ranged from 0.822 to 0.627.

### 3.2. Bivariate Associations between the Derived Factors, Physical Activity, and Time Spent outside

Table 3 presents bivariate associations between the derived factors and between the factors and weekday steps and weekday time spent outside playing. Related to weekday steps, two significant associations of low strength were observed with parental-reported factors, including “neighborhood play with friends” (*r* = 0.199, *p* = 0.022) and “parental PA” (*r* = 0.262, *p* = 0.002), and three significant associations with child-reported factors, one of low strength “tangible parental support” (*r* = 0.222, *p* = 0.008) and two of moderate strength including “general friend support”** (***r* = 0.343, *p* < 0.001**)** and “friends' PA norms”** (***r* = 0.333, *p* < 0.001**)**. One significant association of low strength was observed between time spent outside and parent-reported “neighborhood play with friends” (*r* = 0.281, *p* = 0.002), and three significant associations of low strength were observed between time spent outside and child-reported “active parents” (*r* = 0.188, *p* = 0.036), “friends' PA norms” (*r* = 0.237, *p* = 0.008), and “intangible parental support” (*r* = 0.231, *p* = 0.010). The highest correlation of moderate strength was observed between child-reported “general friend support” and time spent outside playing** (***r* = 0.460, *p* < 0.001**)**. Related to weekend steps (not tabulated), the only significant association observed was with child-reported “tangible parental support”** (***r* = 0.176, *p* = 0.049**)**, and related to weekend time spent outside playing, two significant associations were recorded, one with child-reported “general friend support”** (***r* = 0.213, *p* = 0.020**)** and with child-reported “friends' PA norms”** (***r* = 0.181, *p* = 0.049**)**.

### 3.3. Means of Physical Activity and Time Spent outside Playing across Three Categories of Person with Whom the Child Reported Spending Time outside

Table 4 shows means and 95% confidence intervals of steps and time spent outside playing for each day of measurement by the person with whom the child reported spending time outside. Because of missing data relating to the children's diary where children noted with whom they spend each day outside playing (missing data ranged from 58.4% during weekdays three and four to 42.9% during the Saturday), number of participants for each day is also presented. As only one or two children reported spending time with a parent or with another adult during the six days of measurement and between eight and 11 children reported spending time outside playing on their own, these three categories were merged into one.

The ANOVA analysis for weekday one revealed statistically significant results for both PA [*F*(2,75) = 3.317, *p* = 0.042] and time spent outside playing [*F*(2,79) = 4.210, *p* = 0.018]. Children spending time out with friends recorded higher steps (*p* = 0.042) than children spending time out with their brothers/sisters and higher time outside (*p* = 0.031) than children spending time out alone or with parent/other adult (see Table 4 for mean values). The ANOVA analysis for Saturday also revealed significant results for both PA [*F*(2,75) = 8.744, *p* < 0.001] and time spent outside playing [*F*(2,85) = 6.218, *p* = 0.003]. Children spending time out with friends recorded higher steps than the other two categories (*p* = 0.003 for time out alone or with parent/other adult and *p* = 0.013 for time out with brothers/sisters) and higher time outside (*p* = 0.005) than those spending time out alone or with parent/other adult. During Sunday, a statistically significant difference was revealed for time spent outside playing [*F*(2,82) = 10.438, *p* < 0.001]. Children spending time out with friends spent higher time outside than those spending time out alone or with parent/other adult (*p* < 0.001) and than those spending time out with their brothers/sisters (*p* = 0.031). While, during weekdays two and three, children who reported spending time out with friends also recorded higher mean steps and higher mean time spent outside playing in comparison to children who spent time outside with others, these differences were not statistically significant.

## 4. Discussion

One of the aims of this study was to present evidence of structural validity and reliability of questionnaires assessing parental and friends' correlates of children's PA. Our findings partially support the use of scales used in previous studies. For example, five items to assess parental support used in the studies by Sallis et al. [34] and Trost et al. [23], with an alpha coefficient of 0.78, and the six items relating to general support used in the Jago et al. study [24], with an alpha coefficient of 0.83, were used in the present study. In our study, these items loaded on three different factors, one relating to “general parental support” (parent-reported) and two relating to child-reported tangible and intangible support. Internal consistency of these scales were 0.83, 0.70, and 0.63, respectively. Furthermore, our study confirmed the factor structures of the four items relating to “active parents” in the Jago et al. [24] study, with an *α* coefficient of 0.84, and of the three items relating to “friends' PA norms” with an *α* coefficient of 0.70. The respective reliability coefficients in our study were 0.82 and 0.66. The fact that the structure of these items was confirmed in different studies with diverse samples and language provides further support for their use in future studies.

A unique aspect of the present study was that it used two questionnaires, a parental and a child questionnaire to examine parental and friends' correlates of PA. Both questionnaires contained ten items that were similar and related to parental correlates of PA. Obtaining similar data from both parents and children is important, as children may perceive PA related parenting behaviors differently than that reported by parents. This is illustrated with two related examples in our study. Firstly, while in the parental questionnaire the two items on parental PA and the two items on parental PA with child formed two distinct factors, in the child's questionnaire these four items loaded on a single factor. Conversely, while in the parental questionnaire a single factor containing all parental support items was formed, in the child's questionnaire, two distinct factors were formed, one indicating tangible and the other intangible support. While our findings may be affected by the presence of other items unique to the two questionnaires, they suggest that some parental behaviors may be perceived differently by children. This has implications for future studies as important correlates of PA may not be captured, depending on whom (parent or child) provides the information, and may thus not appropriately be targeted in intervention studies. Our findings address an important gap in parenting PA practices as only a limited number of studies have included measures of both child and parent reports, and more research is needed in this domain [28].

Two scales relating to friends' influences, one in the parental questionnaire (“neighborhood play with friends”) and one in the child questionnaire (“general friend support”) emerged in this study, supporting the important role friends play in children's PA participation [12, 13]. Furthermore, significant associations between similar constructs from the two questionnaires were observed. Parental-reported “general parental support,” “neighborhood play with friends,” and “Parental PA with child” were associated with child-reported “tangible parental support” (*r* = 0.432, *p* < 0.001), “general friend support” (*r* = 0.312, *p* < 0.001), and “active parents” (*r* = 0.201, *p* = 0.016), respectively, thus providing evidence of ecological validity of our data [12]. Internal consistency reliability of six out of the nine factors exceeded the generally accepted level of 0.70, indicating interrelatedness between the items loading in a single factor [44]. However, the three scales that had an *α* lower than 0.70 had only two and three items loading on them, and, according to Cortina [44], scales with fewer items tend to have lower alphas. In this study, we have provided validity and reliability evidence of scales assessing parental and friends' influences in PA, thus addressing a gap in the literature that argues for the development of more multidimensional constructs [11, 29, 30].

Five out of the nine scales in the present study were significantly associated with children's pedometer-determined PA, thus providing evidence of construct validity of the scales developed [30]. The highest associations were observed between PA and child-reported “general friend support” and “friend's PA norms.” Our findings are in agreement with those of Jago et al. [45] who among a sample of children of similar age from the UK found that those who take part in PA with a friend in the neighborhood or at home are more physically active. Additionally, in our study the parental-reported “neighborhood play with friends” scale was also associated with PA. Our evidence suggests that friends influence children's PA levels through a number of mechanisms, including meeting with neighborhood friends in the after-school period to play and friends' positive norms about PA.

Two scales from our questionnaire relating to parental practices were significantly associated with children's PA, the parent-reported “parental PA” and the child-reported “tangible parental support.” In contrast to previous studies that found that parental-reported support for PA among children was significantly related to children's PA [23, 46, 47], our study did not find such an association in the parental questionnaire. While parental encouragement is a strong determinant of children's PA [7], it should be noted that this variable was removed from the parental questionnaire because it loaded on two factors. Nevertheless, in the children's questionnaire, tangible support including transporting and paying to attend a sports club, buying sports related equipment, and watching the child participate, was a significant correlate of children's PA, suggesting that this form of support as perceived by children may be a more important correlate of their activity. One study conducted among children of similar age in the US found that both tangible support and intangible support were associated only with girls' PA participation [22]. In accordance with a study among younger children [48], parental-reported PA was significantly associated with children's PA, suggesting that parental PA modeling may also be targeted in intervention studies to promote children's PA.

Previous studies that have examined both parental and friends' influences on adolescents' PA levels have produced mixed results, with some suggesting that parents only influence children's PA levels [26], while others finding that only friends influence PA in unstructured settings [27]. While, in our study, associations observed with PA were higher in scales relating to friends' influence, our results concur with recent studies that suggest that the more the social support adolescents that they receive from both parents and friends, the higher the likelihood that they will be more active [49] and that future research should address both parental and friends' influences on children's PA [7, 10, 11].

In our study, time spent outside was significantly associated with both weekday (*r* = 0.358, *p* < 0.001) and weekend PA (*r* = 0.214, *p* = 0.026), confirming previous studies that found significant associations between time spent outside and PA participation [21]. Associations of low magnitude were observed between time spent outside and child-reported “active parents” and “intangible parental support” confirming findings from a study conducted in the US that parental support was associated with child outdoor PA [17]. Interestingly, all derived factors relating to friend support were significantly associated with time spent outside, with “general friend support” having the highest association observed in our study. These findings suggest that when promoting time spent outside, friend-related variables should also be targeted including policies that encourage the child to meet with his/her friends to play in the after school period, friends' norms about PA, and friends' own PA behavior.

The above findings may be further enhanced by the observation based on the child's diary that children who reported spending time out with their friends also reported higher PA participation and higher time playing outside in comparison to those children reporting spending time with others. This finding concurs with findings of a recent study conducted in the UK that, for both boys and girls, being outdoors with a friend had the most important influence on the child's moderate to vigorous PA [19]. Targeting time spent outside, in particular with friends, may be an important intervention component in future studies.

While this study contributed to the literature by providing information from both parents and children on social influences on children's PA and time spent outside, a number of limitations should also be acknowledged. First, the relatively small sample size did not allow us to conduct gender-specific analyses or use multivariate analyses to examine the association between predictor and outcome variables. Second, the cross-sectional design precluded us from inferring cause and effect associations between the derived factors, PA, and time spent outside playing. Third, 81.3% of the parents that completed the questionnaire were mothers, and future research should investigate the role of fathers with regard to parental support, PA [7], and time spent outside. Fourth, whereas we have examined parental practices that may be associated with children's PA and time spent outside, we did not investigate the effects of parental styles, that is, the emotional climate created by the practices [28]. Lastly, information relating to time spent outside was obtained by self-report, and the use of objective measures such as Global Positioning System receivers [19] would increase the validity of our data.

## 5. Conclusions

This study provided acceptable evidence of structural validity and adequate reliability of scales assessing parental and friends' influences on children's PA. We have also demonstrated that obtaining information relating to parental and friends' influences on children's PA from both parents and their children may provide a more complete picture of possible influences. This is because children may perceive parental behavior differently than that reported by their parents. Stronger associations were observed with friends' influences rather than parental practices, suggesting that interventions studies among children of this age should also target friendship groups and time spent outside playing to improve program effectiveness.

## Figures and Tables

**Table 1:** Principal component analysis of the items in the parental questionnaire assessing parental and friends' influence on children's physical activity.

	General parental support	Neighborhood play with friends	Parental PA	Parental PA with child
I pay for my child to attend a sports club (e.g. soccer, swimming)	.893			
I transport my child to a sports club in the after school period	.847			
I buy sports clothes and other sports equipment related to the sports that my child engages in	.763			
My child attends a sports club with a friend	.671			
I talk to my child about the benefits of physical activity	.607			

My child meets some friends in the afternoon to play outside in the neighborhood		.860		
There are children of the same age of my child in the neighborhood for him/her to go out and play		.841		
Near our house there are houses of my child's friends where my child can walk on his/her own to meet friends		.829		
My child visits the houses of some friends for play		.369		

During weekdays, how much time do you spend being physically active (e.g. walking, gym)?			.909	
During weekends, how much time do you spend being physically active (e.g. walking, gym)?			.889	

I take part in physical activity with my child during weekdays (e.g. walking, cycling)				.849
I take part in physical activity with my child during weekends (e.g., walking, cycling)				.844

Eigenvalues	3.025	2.394	1.708	1.545
Percentage of variance explained by each factor	23.271	18.414	13.138	11.882
Cronbach's alpha of each factor	0.828	0.768	0.767	0.650

**Table 2:** Principal component analysis of the items in the children's questionnaire assessing parental and friends' influence on children's physical activity.

	Active parents	General friend support	Tangible parental support	Friend's PA norms	Intangible parental support
My parents or other adults who live with me, take part in physical activity with me during weekends	.827				
My parents or other adults who live with me, take part in physical activity with me during weekdays	.809				
My parents or other adults who live with me, take part in lots of physical activity during weekends	.777				
My parents or other adults who live with me, take part in lots of physical activity during weekdays	.663				

My friends, that I spend time with after school, encourage me to be physically active		.797			
My friends, that I spend time with after school, engage in physical activity with me in the afternoon		.736			
I go to a friend's house in the afternoon to do some physical activity		.718			
My friends, that I spend time with after school, go out in the afternoon to do some physical activity/sport		.706			

My parents or other adults who live with me, transport me to a sports club in the after school period			.825		
My parents or other adults who live with me, pay for me to attend a sports club (e.g. soccer, swimming)			.780		
My parents or other adults who live with me, buy for me sports clothes and other equipment relating to the sports I engage in (e.g. balls)			.603		
My parents or other adults who live with me, watch me while engaging in physical activity (e.g. playing football, swimming)			.511		

My friends believe that it's a very good thing to engage in physical activity (e.g. running or playing a sport)				.876	
My friends believe that it's a very good thing to attend sports clubs in the afternoon (e.g. soccer, swimming)				.692	
My friends participate in a lot of physical activities (e.g. running or playing a sport)				.645	

My parents or other adults who live with me, encourage me to be physically active					.782
My parents or other adults who live with me, talk to me about the benefits of physical activity					.751

Eigenvalues	2.676	2.389	2.210	1.937	1.641
Percentage of variance explained by each factor	15.743	14.056	12.998	11.396	9.655
Cronbach's alpha of each factor	0.822	0.748	0.700	0.661	0.627

**Table 3:** Bivariate associations between the derived factors, physical activity, and time spent outside.

	General parental support_PR	Neighborhood play with friends_PR	Parental PA_PR	Parental PA with child_PR	Active parents_CR	General friend support_CR	Tangible Parental Support_CR	Friends' PA norms_CR	Intangible Parental support_CR	Weekday steps	Weekday time outside playing
General parental support_PR	1.000	0.249^*∗∗*^ (*p* = 0.003)	0.161 (*p* = 0.053)	0.200^*∗*^ (*p* = 0.016)	−0.104 (*p* = 0.214)	−0.024 (*p* = 0.771)	0.432^*∗∗∗*^ (*p* = 0.000)	0.053 (*p* = 0.527)	0.158 (*p* = 0.058)	0.120 (*p* = 0.169)	−0.094 (*p* = 0.300)
Neighborhood play with friends_PR		1.000	0.136 (*p* = 0.104)	0.081 (*p* = 0.332)	0.052 (*p* = 0.535)	0.312^*∗∗∗* ^ (*p* = 0.000)	0.135 (*p* = 0.107)	0.187^*∗*^ (*p* = 0.025)	0.148 (*p* = 0.077)	0.199^*∗*^ (*p* = 0.022)	0.281^*∗∗*^ (*p* = 0.002)
Parental PA_PR			1.000	0.219^*∗∗*^ (*p* = 0.008)	0.036 (*p* = 0.667)	0.183^*∗*^ (*p* = 0.028)	0.014 (*p* = 0.866)	0.097 (*p* = 0.246)	−0.011 (*p* = 0.899)	0.262^*∗∗*^ (*p* = 0.002)	0.044 (*p* = 0.626)
Parental PA with child_PR				1.000	0.201^*∗*^ (*p* = 0.016)	0.006 (*p* = 0.944)	0.078 (*p* = 0.356)	−0.018 (*p* = 0.833)	0.048 (*p* = 0.570)	0.057 (*p* = 0.518)	−0.004 (*p* = 0.967)
Active parents_CR					1.000	0.313^*∗∗∗*^ (*p* = 0.000)	0.347^*∗∗∗*^ (*p* = 0.000)	0.262^*∗∗*^ (*p* = 0.001)	0.181^*∗*^ (*p* = 0.025)	0.144 (*p* = 0.088)	0.188^*∗*^ (*p* = 0.036)
General friend support_CR						1.000	0.209^*∗∗*^ (*p* = 0.009)	0.369^*∗∗∗*^ (*p* = 0.000)	0.234^*∗∗*^ (*p* = 0.003)	0.343^*∗∗∗*^ (*p* = 0.000)	0.460^*∗∗∗*^ (*p* = 0.000)
Tangible Parental Support_CR							1.000	0.333^*∗∗∗*^ (*p* = 0.000)	0.400^*∗∗∗*^ (*p* = 0.000)	0.222^*∗∗*^ (*p* = 0.008)	0.068 (*p* = 0.450)
Friends' PA norms_CR								1.000	0.347^*∗∗∗*^ (*p* = 0.000)	0.333^*∗∗∗*^ (*p* = 0.000)	0.237^*∗∗*^ (*p* = 0.008)
Intangible Parental support_CR									1.000	0.140 (*p* = 0.097)	0.231^*∗∗*^ (*p* = 0.010)
Weekday steps										1.000	0.358^*∗∗∗*^ (*p* = 0.000)
Time outside playing											1.000

(i) PR = parent-reported; CR = child-reported; PA = physical activity.

(ii) ^*∗*^*p* < 0.05, ^*∗∗*^*p* < 0.01, ^*∗∗∗*^*p* < 0.001.

(iii) *n* = 133 for associations between PR scales and steps; *n* = 124 for associations between PR scales and time outside playing; *n* = 141 for associations between CR scales and steps; *n* = 125 for associations between CR scales and time outside playing.

**Table 4:** Means and 95% confidence intervals of steps and time spent outside playing (minutes) for each day by the person with whom the child reported spending time outside.

	Friend(s)	Brother(s) or sister(s)	Alone or parent or other adult
Weekday 1			
Steps/day (*n* = 78)	18,068^a^ (16,354–19,782)^b^	14,593 (12,713–16,473)	16,924 (12,659–21,190)
Time spent outside playing (*n* = 82)	135.0^a^ (104.4–165.6)^b^	96.7 (65.2–128.1)	54.5 (26.3–82.8)
Weekday 2			
Steps/day (*n* = 68)	17,248 (15,509–18,988)	15,799 (13,627–17,970)	14,761 (13,097–16,425)
Time spent outside playing (*n* = 73)	120.8 (92.0–149.6)	114.0 (76.8–151.2)	70.9 (16.6–125.2)
Weekday 3			
Steps/day (*n* = 60)	16,868 (14,624–19,112)	14,409 (11,533–17,285)	14,002 (10,930–17,074)
Time spent outside playing (*n* = 64)	144.5 (107.3–181.8)	111.7 (68.0–155.3)	83.1 (31.7–134.4)
Weekday 4			
Steps/day (*n* = 52)	15,013 (12,799–17,227)	16,736 (12,867–20,605)	16,617 (13,280–19,953)
Time spent outside playing (*n* = 64)	127.2 (89.8–164.7)	104.3 (70.9–137.6)	64.3 (29.7–98.8)
Saturday			
Steps/day (*n* = 78)	16,804 (15,229–18,379)	12,896 (10,643–15,149)	9,708 (6,465–12,951)
Time spent outside playing (*n* = 88)	169.0 (144.8–193.2)	133.3 (101.7–165.0)	70.0 (31.8–108.2)
Sunday			
Steps/day (*n* = 74)	14,477 (12,477–16,476)	11,424 (8,978–13,870)	14,647 (10,980–18,314)
Time spent outside playing (*n* = 85)	189.6 (162.0–217.1)	131.5 (93.7–169.4)	67.5 (45.8–89.2)

(i) ^a^Mean values.

(ii) ^b^95% confidence intervals.
